# Clinical analysis and follow up study of chronic granulomatous disease with neonatal onset

**DOI:** 10.3389/fped.2026.1874398

**Published:** 2026-07-09

**Authors:** Dongwei Zhang, Xuehua Xu, Huifeng Fan, Gan Zhou, Wenyan Li, Diyuan Yang, Peiqiong Wu, Gen Lu

**Affiliations:** Guangzhou Women and Children’s Medical Center, Guangzhou Medical University, Guangzhou, China

**Keywords:** chronic granulomatous disease, clinical, *CYBB*, neonatal, prognosis

## Abstract

**Objective:**

To address the existing gap in knowledge regarding the small subset of chronic granulomatous disease (CGD) patients who present during the neonatal period, this study aimed to characterize their clinical features, genetic profiles, and long-term prognosis.

**Methods:**

This study retrospectively selected CGD patients with neonatal onset who were diagnosed using neutrophil respiratory burst tests and genetic analysis at the Guangzhou Women and Children's Medical Center, Guangzhou Medical University, between January 2018 and September 2024.

**Results:**

This study included nine male patients diagnosed with CGD presenting with neonatal onset. The median age at onset was 20 days (7–23 days). Pneumonia was observed in eight patients (88.9%), with Aspergillus species detected in five of these cases (62.5%). The stimulation index (SI) in the neutrophil oxidative burst assay was significantly reduced. All nine patients exhibited hemizygous variants in the *CYBB* gene. The median hospital stay was 42 days (15.5–48 days). Three patients died due to disease progression during their initial hospitalization. Regarding long-term prognosis, one patient was lost to follow-up at six months of age, and three patients died from severe infections during infancy. The remaining two patients underwent hematopoietic stem cell transplantation (HSCT) and achieved complete clinical remission.

**Conclusion:**

Patients with CGD presenting in the neonatal period predominantly harbor mutations in the *CYBB* gene, which are associated with severe clinical manifestations. Aspergillus species are the most common pathogens in these patients. Although the prognosis is generally poor, it may be improved by hematopoietic stem cell transplantation (HSCT).

## 1 Introduction

Chronic granulomatous disease (CGD) is a primary immunodeficiency resulting from a defect in the phagocytic function of the innate immune system, caused by mutations in genes encoding subunits of the nicotinamide adenine dinucleotide phosphate (NADPH) oxidase enzyme complex (1, 2). The genes involved include *CYBB*, *CYBA*, *NCF1*, *NCF2*, *NCF4*, and *CYBC1* (2). Children with CGD suffer from recurrent, life-threatening bacterial and fungal infections and may develop infectious or inflammatory complications. Although the clinical characteristics and diagnostic indicators of CGD in children are relatively well understood, CGD with neonatal onset is even rarer, with only a limited number of case reports and literature reviews available (3, 4). In this study, we conducted a clinical analysis of nine CGD patients with neonatal onset to elucidate the disease's characteristics, diagnostic approaches, and genetic mutation patterns. Our aim is to enhance clinicians’ understanding of this condition, thereby promoting early diagnosis and improving prognosis.

## 2 Materials and methods

### 2.1 Study population

The study retrospectively selected CGD patients with neonatal onset who were diagnosed using neutrophil respiratory burst tests and genetic analysis at Guangzhou Women and Children's Medical Center, Guangzhou Medical University, from January 2018 to September 2024. This research was approved by the hospital's Ethics Committee (approval number 2025048A01).

### 2.2 Clinical data collection

#### 2.2.1 Data collection

Review electronic medical records to gather information on patients’ gender, gestational age, birth weight, age at illness onset, initial symptoms, clinical manifestations, severity and duration of illness, any adverse family history such as stillbirth or miscarriage, and whether respiratory support with a ventilator is required. Collect results of cellular and humoral immunity tests, neutrophil respiratory burst tests, sputum, blood, and bronchoalveolar lavage fluid (BAF) cultures along with drug sensitivity tests; radiological findings from chest x-rays or chest computed tomography (CT) scans; and the timing and results of genetic testing. Additionally, collect details on the treatment plan, prognosis, and follow-up outcomes. Follow-up methods include reviewing outpatient re-examination records and conducting telephone follow-ups. Follow-up indicators encompass the timing of follow-up, the child's survival status, medication adherence, presence of recurrent infections, whether hematopoietic stem cell transplantation (HSCT) has been performed, and the child's growth and developmental status.

#### 2.2.2 Diagnosis of CGD

CGD typically presents with recurrent and severe bacterial and/or fungal infections. Diagnostic methods for CGD include the nitroblue tetrazolium (NBT) reduction test, dihydrorhodamine (DHR) flow cytometry assay, protein expression analysis, and gene sequencing (2). Currently, our hospital employs the DHR assay to evaluate neutrophil respiratory burst function.

#### 2.2.3 Pathogen detection

The detection of bacterial or fungal pathogens is conducted through blood culture, sputum culture, bronchoalveolar fluid (BAF) culture, or next-generation sequencing (NGS) of blood and BAF samples. Respiratory viruses and Mycoplasma pneumoniae are identified using real-time PCR of nasopharyngeal swabs or NGS of BAF.

### 2.3 Statistical analysis

Continuous variables were first tested for normality using the Shapiro–Wilk test. Normally distributed continuous variables are presented as mean ± standard deviation (SD), while non-normally distributed continuous variables are presented as median (interquartile range, IQR). Categorical variables are summarized as numbers (percentages).

## 3 Results

### 3.1 Birth and family history

Over a six-year period, nine male patients with neonatal-onset CGD were admitted to our hospital. The birth and family histories of these patients are summarized in Table 1. All patients were male. Five patients (55.56%) had a positive family history, which included early deaths of male siblings, stillbirths, and early deaths of male children born to the maternal grandmother.

**Table 1:** Birth and family history of patients with chronic granulomatous disease presenting in the neonatal period.

Patient	Gender	Conception method	Premature infant (Y/N)	Delivery mode	Birth weigh（kg）	Family history^a^ （Y/N）
Case 1	Male	Natural Conception	N	Cesarean section	2.85	Y
Case 2	Male	Natural Conception	N	Vaginal Delivery	2.85	N
Case 3	Male	Natural Conception	Y	Cesarean section	2	Y
Case 4	Male	Natural Conception	N	Vaginal Delivery	2.9	N
Case 5	Male	Natural Conception	N	Vaginal Delivery	3.4	N
Case 6	Male	Natural Conception	Y	Cesarean section	2.4	N
Case 7	Male	Natural Conception	N	Vaginal Delivery	3.4	Y
Case 8	Male	*In vitro* fertilization	N	Cesarean section	3.5	Y
Case 9	Male	Natural Conception	N	Vaginal Delivery	2.9	Y

a The family history include the death of male siblings at a young age, stillbirths, and the death of male children born to the maternal grandmother at a young age in the family.

Y, Yes; N, No.

### 3.2 Clinical and laboratory characteristics

**Figure 1:**
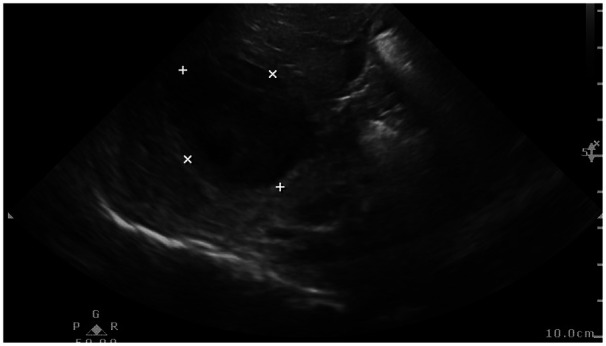
Abdominal ultrasound of patient 7 revealed a hypoechoic mass in liver segments S5 and S6, measuring approximately 42 × 33 × 54 mm. The mass exhibited well-defined borders, irregular internal walls, and areas of partial liquefaction appearing as dark regions.

**Figure 2:**
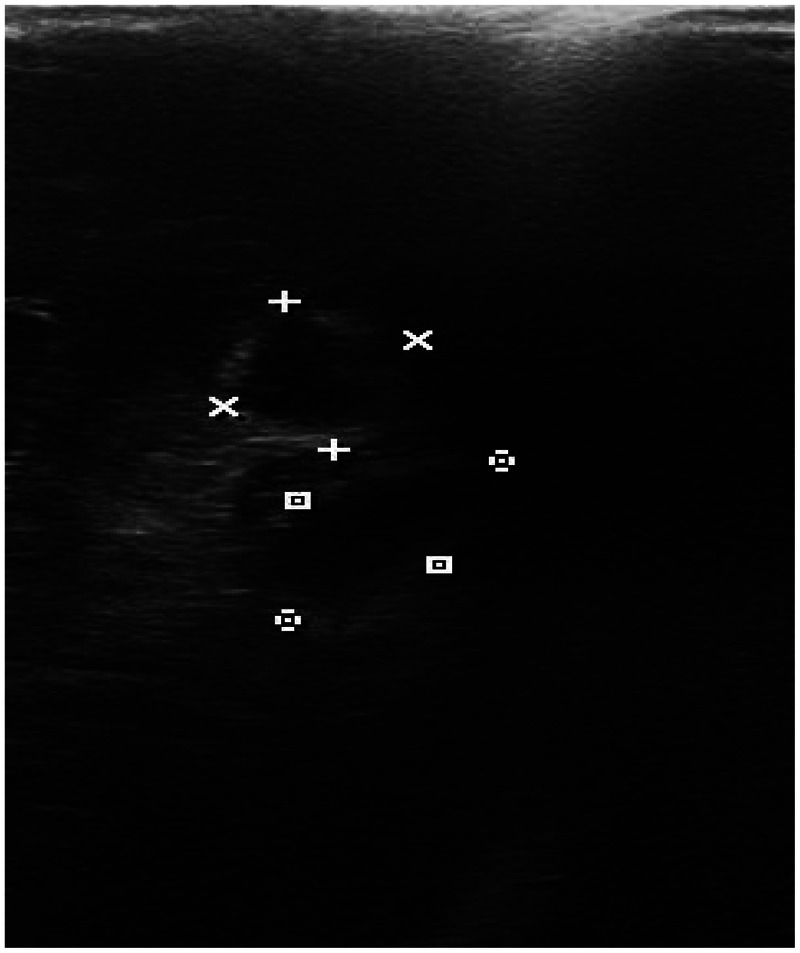
Cranial ultrasound of patient 7 revealed two abnormal echoes in the left occipital lobe parenchyma, measuring approximately 11 × 10 mm and 15 × 8 mm, respectively. The lesions exhibited relatively well-defined margins, were surrounded by a slightly hyperechoic rim, and showed reduced internal echogenicity.

The median age of onset for all patients was 20 days (7–23 days). The most common clinical symptoms were tachypnea, fever, and cough, occurring in 100%, 88.9%, and 77.8% of patients, respectively. Four patients exhibited moist rales on auscultation. Regarding clinical diagnosis, eight patients were diagnosed with pneumonia. Among these, four developed lung abscesses, and five exhibited extrapulmonary manifestations, including sepsis in two patients, skin abscesses in two patients, a liver abscess in one patient (Figure 1), and a brain abscess in one patient (Figure 2), among others.

All patients exhibited significantly elevated white blood cell counts, with a mean value of 30.66 ± 9.24 × 10^9^/L. The mean neutrophil percentage was 58.33 ± 11.83%. C-reactive protein levels were markedly increased in all patients, averaging 88.61 ± 61.19 mg/L. Procalcitonin levels were measured in five patients, all of whom showed elevated values, with a mean of 2.50 ± 2.89 ng/mL. Hypoalbuminemia was observed in seven patients, including two with albumin levels below 25 g/L. Other biochemical parameters, including liver function, renal function, and coagulation function, remained essentially within normal ranges (Table 2).

**Table 2:** Laboratory characteristics of chronic granulomatous disease with neonatal onset at admission.

Laboratory tests	Measured values
Blood routine examination (normal range)	
C-reactive protein (≤1.6 mg/L)	88.61 ± 61.19
White blood cell (7.0–20.0 10^9^/L)	30.66 ± 9.24
Neutrophil ratio (7–56%)	58.33 ± 11.83
Hemoglobin (135–195 g/L)	90.00(86.00–105.50)
Blood platelet (183–614 10^9^/L)	461.89 ± 191.76
Liver and kidney function (normal range)	
Alanine aminotransferase (6–33 U/L)	47.56 ± 37.94
Aspartate aminotransferase (22–71 U/L)	48.89 ± 29.48
Albumin (40–55 g/L)	32.48 ± 6.58
Lactic dehydrogenase (159–322 U/L)	341.11 ± 100.12
Total bilirubin (2–17 umol/L)	7.20 (4.10–10.75)
Creatinine (18–97 umol/L)	20.89 ± 7.20
Cystatin C (0.55–1.05 mg/L)	1.22 ± 0.23
Coagulation function(normal range)	
Prothrombin time (11–15 s)	14.15 ± 1.01
Activated partial thromboplastin time (28–45 s)	40.75 ± 4.77
International normalized ratio (0.8–1.5)	1.09 ± 0.10
Neutrophil respiratory burst test (normal range)	
Phosphate buffered saline(PBS)(<10%)	67.69 ± 18.18
Phorbol 12-myristate 13-acetate (PMA) (>90%）	94.40 (86.48–98.03)
Stimulation index(SI) (>100）	2.84 (1.51–21.8)

### 3.3 Microbiological investigation

Of the eight neonatal pneumonia patients, six had definitive respiratory pathogen results. Among these, Aspergillus fumigatus was detected in five patients (62.5%), Pseudomonas aeruginosa (Pa) in two patients (25%), Aspergillus flavus in one patient, Mycoplasma pneumoniae in one patient, and respiratory syncytial virus in one patient. Among the five patients diagnosed with Aspergillus fumigatus infection, one was confirmed by BAF culture, three by NGS of BAF, and one by sputum culture. Two or more respiratory pathogens were detected simultaneously in three patients (37.5%).

### 3.4 Chest imaging

**Figure 3:**
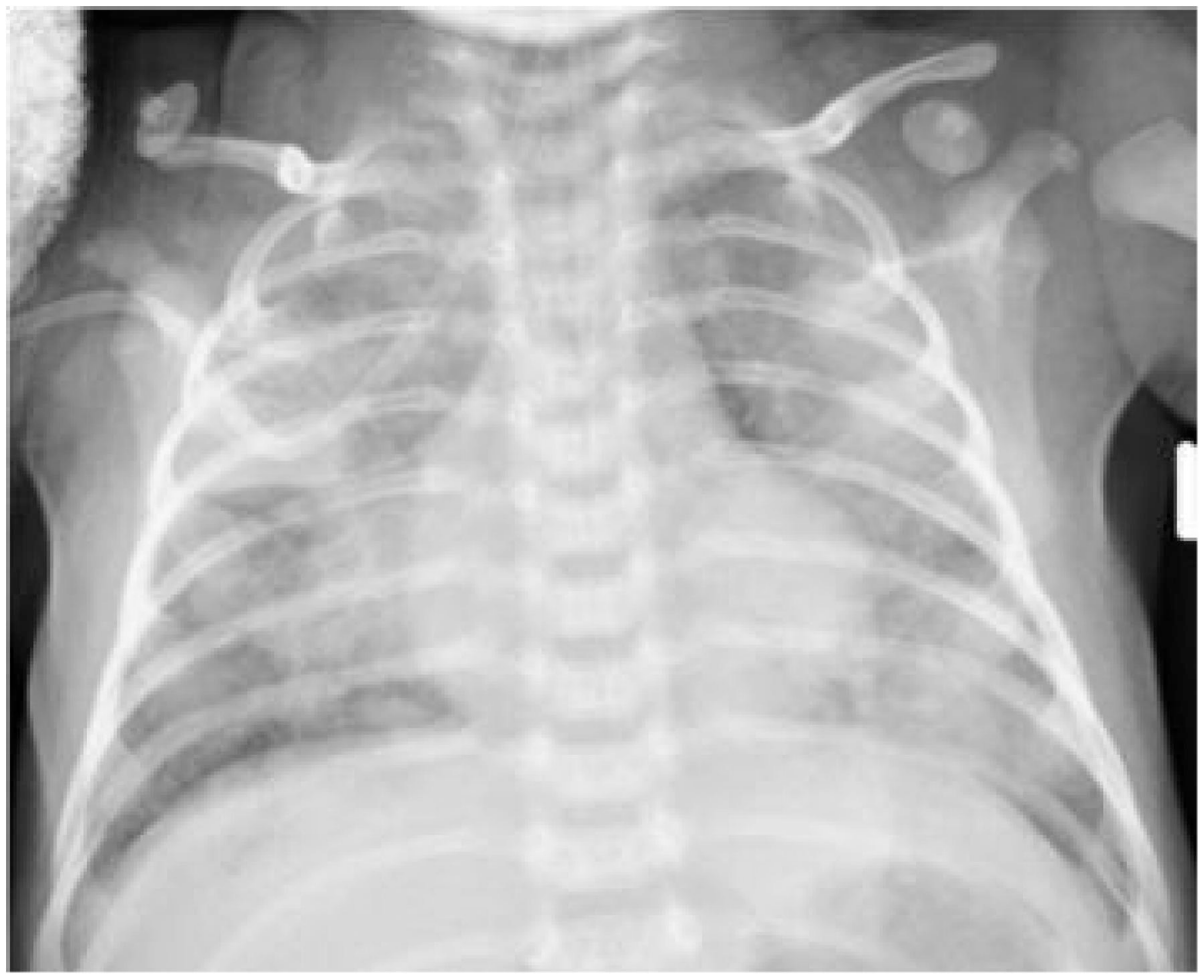
Chest x-ray reveals multiple patchy opacities in both lungs.

**Figure 4:**
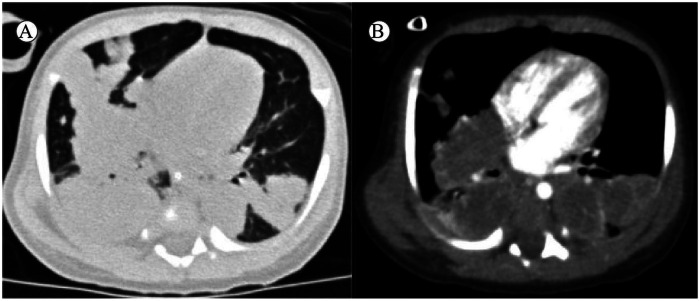
**(A)** chest CT scans demonstrate multiple patchy, large consolidations and mass opacities in both lungs. **(B)** Contrast-enhanced imaging reveals prominent, non-enhancing, hypodense necrotic changes within the lesions.

All nine patients completed chest x-ray examinations; eight showed signs of pneumonia, while one appeared normal. Among the eight pneumonia patients, chest x-rays consistently revealed multiple patchy opacities involving several lung lobes in seven patients (87.5%) (Figure 3). The remaining patient exhibited a small amount of exudate in bilateral lung lobes. All eight pneumonia patients underwent contrast-enhanced chest CT scans due to severe chest x-ray findings or unsatisfactory treatment progress. Chest CT scans demonstrated multiple patchy, large consolidations and mass opacities in both lungs in seven patients (87.5%), with some lesions appearing coalescent and ill-defined. Contrast-enhanced imaging revealed prominent non-enhancing hypodense necrotic changes within the lesions in three patients, clinically suggestive of lung abscess (Figure 4). Chest CT also revealed a cavitary lesion consistent with lung abscess in one patient. Of these four patients with lung abscess, Aspergillus fumigatus was identified in three, while Pa was detected in one. None of the patients exhibited hilar or mediastinal lymphadenopathy.

### 3.5 Immunological evaluation

The results of cellular and humoral immunity tests in all nine patients showed no significant abnormalities. The stimulation index (SI) in the neutrophil oxidative burst assay was consistently below 100 in all patients (reference range: >100), with a median of 2.84 (1.51–21.8).

### 3.6 Genetic test results

All nine patients exhibited hemizygous variants in the *CYBB* gene. Among these variants, four were maternally inherited, four were spontaneous mutations, and one patient did not undergo parental genetic testing. Regarding the amino acid alterations, the cohort included two patients each with deletions, frameshift mutations, nonsense mutations, and splice-site mutations, along with one case of a missense mutation (Table 3).

**Table 3:** Genetic test results of chronic granulomatous disease patients with neonatal onset.

Patient	Gene	Variant Type	Nucleotide Alteration	Amino Acid Variation	Variant Origin	Inheritance Pattern
Case1	*CYBB*	Hemizygous variant	Deletion	-	-^a^	XR
Case2	*CYBB*	Hemizygous variant	c.668–671delAGCinsCAAG	p.A224Kfs, Frameshift mutation	Spontaneous mutation	XR
Case3	*CYBB*	Hemizygous variant	NM-000397.3:c.1038delT	p.Glu347fs, Frameshift mutation	Mother	XR
Case4	*CYBB*	Hemizygous variant	c.466G＞A	p.Ala156Thr, Missense mutation	Spontaneous mutation	XR
Case5	*CYBB*	Hemizygous variant	c.484–8T＞G	Splice-site mutation	Mother	XR
Case6	*CYBB*	Hemizygous variant	c.1459C＞T	p.Gln487Ter, Nonsense mutation	Spontaneous mutation	XR
Case7	*CYBB*	Hemizygous variant	Deletion	-	Mother	XR
Case8	*CYBB*	Hemizygous variant	c.217C＞T	p.R73X, Nonsense mutation	Spontaneous mutation	XR
Case9	*CYBB*	Hemizygous variant	c.1586 + 3A＞T	Splice-site mutation	Mother	XR

a Gene not tested in parents.

XR X-linked recessive inheritance.

### 3.7 Treatment, outcome, and follow-Up

Intravenous immunoglobulin therapy was administered to four patients. Non-invasive ventilator support was used in five patients, while three required endotracheal intubation for mechanical ventilation. All patients received anti-infective treatment. Empirical antibiotic therapy was initiated early during hospitalization based on inflammatory markers and clinical manifestations, prior to obtaining etiological evidence. The antimicrobial regimen was subsequently adjusted according to pathogen identification. Except for two patients, all required combination therapy with more than two antibiotics. Specific antibiotic regimens and treatment durations are detailed in Table 4. The median hospital stay for all patients was 42 days (15.5–48 days).

**Table 4:** Treatment and prognosis of chronic granulomatous disease in patients with neonatal onset.

Patient	Clinical manifestations	Microbiological investigation	Antibiotic (course, days)	Length of IMV (days)	Length of hospital stay (days)	Follow up and long-term prognosis
Case 1	Sepsis	**-**	Amoxicillin (14 d)	**-**	14	Died at 13 months of age
	Skin abscess					
	Enteritis					
Case 2	Pneumonia	Aspergillus fumigatus,	Cefoperazone/sulbactam （8 d）	-	75	Lost follow-up at six months of age
	Lung abscess	MP	Azithromycin（5 d）			
			Imipenem （12 d）			
			Linezolid (10 d)			
			Amphotericin B （36 d）			
Case 3	Pneumonia	-	Cefoperazone/sulbactam （14 d）	-	15	HSCT at 2 years old
	NEC					Healthy survival
Case 4	Pneumonia	Aspergillus fumigatus,	Cefotaxime (13 d)	7	44	Died due to disease progression
			Voriconazole (21 d)			
Case 5	Pneumonia	Aspergillus fumigatus	Meropenem (33 d)	28	44	Died at 18 months of age
	Lung abscess		Voriconazole (30 d)			
Case 6	Pneumonia	Aspergillus fumigatus	Cefoperazone/sulbactam（36 d）	-	52	Died at 16 months of age
	Skin abscess	RSV	Voriconazole (26 d)			
		*Pa*				
Case 7	Pneumonia	Aspergillus fumigatus	Meropenem (9 d)	7	16	Died due to disease progression
	Sepsis	Aspergillus flavus	Vancomycin（15 d）			
	Lung abscess	Staphylococcus epidermidis	Voriconazole (10 d)			
	Brain abscess					
	Liver abscess					
	Preseptal cellulitis					
Case 8	Pneumonia	**-**	Piperacillin-tazobactam（21 d）	-	28	Died due to disease progression
	Umbilical infection		Vancomycin（10 d）			
Case 9	Pneumonia	*Pa*	Piperacillin-tazobactam（8 d）	-	42	HSCT at 5 years old
	Lung abscess		Ceftriaxone (17 d)			Healthy survival
			Meropenem (16 d)			
			Vancomycin（33 d)			

IMV, invasive mechanical ventilation; MP mycoplasma pneumoniae; NEC, neonatal necrotizing enterocolitis; RSV respiratory syncytial virus; HSCT hematopoietic stem cell transplantation; *Pa* Pseudomonas aeruginosa; -, None.

During the initial hospitalization period, three patients died due to disease progression. After discharge, the six surviving patients were prescribed trimethoprim-sulfamethoxazole for bacterial infections and itraconazole for fungal infections. One patient was lost to follow-up at six months of age. Three patients succumbed to severe infections during infancy. The remaining two patients underwent HSCT at two and five years of age, respectively, achieving complete clinical remission.

## 4 Discussion

Chronic granulomatous disease (CGD) is a primary immunodeficiency caused by a defect in the phagocytic function of the innate immune system (1). Neutrophils from patients with CGD exhibit deficiencies in neutrophil extracellular traps, autophagy, and apoptosis (2). The diagnosis of CGD is based on clinical manifestations, abnormal neutrophil function testing, and genetic analysis. CGD is relatively uncommon in children and exceedingly rare in neonates (1, 2). Severe pediatric CGD typically presents early, between 9 and 14 months of age, with diagnosis occurring between 2.1 and 4.9 years (5). Diagnosis in neonates is increasing due to greater awareness and more widespread testing. In this study, we present a series of CGD patients with neonatal onset. In our case series, more than half of the patients had a positive family history, including the death of male siblings at a young age, stillbirths, and the death of male children born to the maternal grandmother at a young age. This positive family history heightened our suspicion of an underlying immunodeficiency. All patients were clinically diagnosed based on family history, clinical manifestations, and abnormal neutrophil function, with subsequent confirmation through genetic testing.

The majority of patients in our study presented with pulmonary infections, followed by cutaneous infections and sepsis as the next most common clinical manifestations. This pattern aligns with previous reports in the literature (3, 4). Among the eight patients with pulmonary infections, four developed lung abscesses: three cases were caused by Aspergillus fumigatus, and one by Pseudomonas aeruginosa (Pa). This presentation differs significantly from the typical pulmonary manifestations observed in pediatric CGD patients, where pulmonary nodules and consolidation are the most common radiographic findings, followed by mass lesions (6). Therefore, the presence of lung abscesses may serve as an important diagnostic indicator for neonatal immunodeficiency disorders such as CGD.

Aspergillus species, particularly Aspergillus fumigatus, and Burkholderia species are the most prevalent pathogens in pediatric patients with CGD (6). Patients with CGD exhibit similar respiratory pathogen profiles regardless of whether disease onset occurs in the neonatal period or later in childhood. Aspergillus species remain the predominant infectious agents across all age groups, with only minor differences observed in secondary respiratory pathogens (3, 6). In our case series, five patients were found to have concurrent Aspergillus fumigatus infections. Notably, although Pseudomonas aeruginosa (Pa) is not typically considered a common pathogen in pediatric CGD patients, it was identified in two patients in our study: one co-infected with Aspergillus species and the other as the sole pathogen. While Pa is relatively uncommon among Chinese pediatric CGD patients, it appears to be more prevalent among Egyptian CGD patients (6, 7). These variations in the microbiological spectrum may be associated with regional environmental factors. These findings suggest that Aspergillus infection serves as a diagnostic indicator for CGD with neonatal onset and underscore the importance of vigilance for uncommon pathogens.

The assessment of neutrophil respiratory burst function is a highly sensitive and rapid method for diagnosing CGD, offering greater speed and practicality compared to genetic testing. Historically, the neutrophil respiratory burst function test was considered the gold standard for CGD diagnosis. However, subsequent research has shown that the dihydrorhodamine (DHR) assay can produce false-positive results due to the influence of certain medications, such as acetaminophen and mesalazine (8, 9). Additionally, abnormalities in the DHR assay may also result from myeloperoxidase (MPO) deficiency (10). In this study, patients were clinically suspected of immunodeficiency based on their clinical manifestations and etiological findings, prompting early evaluation of immune function. The results indicated that all patients exhibited normal humoral and cellular immunity, while the neutrophil respiratory burst test revealed significantly reduced activity. Therefore, the neutrophil respiratory burst assay is instrumental in facilitating the early diagnosis of CGD.

Genetic testing not only facilitates the molecular diagnosis of CGD but also enables the assessment of disease severity. The clinical course of CGD is influenced not only by the specific type of gene mutation but also by the mutation mode. For example, mutations in the *CYBB* gene are generally more severe, present earlier in life, and are associated with higher mortality (11). In our case series, all patients exhibited mutations in the *CYBB* gene. This proportion significantly exceeds the reported 58%–60% incidence of *CYBB* gene mutations among CGD patients with neonatal onset in the literature (3, 4). This discrepancy may be attributed to ethnic differences among the studied populations. In our series, two patients exhibited deletions in the *CYBB* gene. One of these patients experienced clinical improvement during the neonatal period, while the other progressed to multiple organ failure. Additionally, two patients with nonsense mutations showed partial clinical improvement following treatment. Due to the limited number of patients and the presence of up to five different mutation types, it is challenging to assess the correlation between mutation mode and disease severity. This limitation underscores the need for future multicenter studies to further investigate the relationship between gene mutations and clinical outcomes during the neonatal period.

The management of patients with CGD presenting in the neonatal period poses significant challenges. Compared with classical pediatric-onset CGD cohorts, our neonatal-onset CGD patients had a higher proportion of severe disease and a worse prognosis (6). In our case series, despite comprehensive anti-infective therapy, fewer than half of the patients achieved complete remission during their neonatal hospitalization. The remaining patients either showed partial improvement or experienced disease progression, with a median hospital stay of 42 days. Similar durations of hospitalization and treatment responses have been reported in other case reports (3). Regarding long-term prognosis, hematopoietic stem cell transplantation (HSCT) remains the only curative therapy for individuals with CGD (12). In our series, both patients who underwent HSCT achieved asymptomatic survival, whereas the three patients who did not receive HSCT died during infancy. This outcome was influenced by complex factors, including severe infections and the lack of suitable donor matches. Indeed, HSCT has demonstrated excellent outcomes in Chinese pediatric CGD patients, with a 3-year post-transplant survival rate of 87.5% reported in a multicenter study (13). Given the significant impact of HSCT on long-term prognosis, meticulous management of neonatal-onset CGD prior to transplantation is essential, including prolonged antibiotic prophylaxis to prevent infections. Furthermore, with the advent of lower-toxicity conditioning regimens, HSCT is now considered a valid therapeutic option early in the disease course rather than solely as salvage therapy (12). The age at which our two patients underwent HSCT was significantly older than that reported in other studies of neonatal-onset CGD patients (4, 14). When feasible, HSCT should be performed as early as possible in patients with neonatal-onset CGD.

Several limitations of this study should be acknowledged. First, the sample size is small, comprising only nine patients, which inevitably limits the statistical power and precludes definitive subgroup comparisons. Second, this is a single-center study. Larger, multicenter studies are needed to further validate our observations and enable more robust statistical analyses.

## 5 Conclusion

CGD with neonatal onset is predominantly caused by mutations in the *CYBB* gene and is characterized by severe clinical manifestations. Aspergillus species are the most prevalent pathogens in CGD patients with neonatal onset. While diagnosis is relatively straightforward, treatment remains challenging, and the long-term prognosis is poor. Early diagnosis and HSCT are the most viable approaches to improving outcomes.

## Data Availability

The raw data supporting the conclusions of this article will be made available by the authors, without undue reservation.
